# Genome-Wide Identification and Analysis of the NAC Transcription Factor Gene Family in Garden Asparagus (*Asparagus officinalis*)

**DOI:** 10.3390/genes13060976

**Published:** 2022-05-30

**Authors:** Caifeng Li, Jingyang Zhang, Qianqian Zhang, Ang Dong, Qiuhong Wu, Xingyu Zhu, Xuli Zhu

**Affiliations:** 1 Center for Computational Biology, College of Biological Sciences and Technology, Beijing Forestry University, Beijing 100083, China; fancykoi@163.com (C.L.); awayzqq@163.com (Q.Z.); fantasys05227@gmail.com (A.D.); hong1481503435@163.com (Q.W.); zxy1716429235@126.com (X.Z.); 2 Tandon School of Engineering, New York University, New York, NY 11201, USA; jz2807@nyu.edu; 3 National Engineering Laboratory for Tree Breeding, Beijing Forestry University, Beijing 100083, China; 4 Key Laboratory of Genetics and Breeding in Forest Trees and Ornamental Plants, Beijing Forestry University, Ministry of Education, Beijing 100083, China; 5 The Tree and Ornamental Plant Breeding and Biotechnology Laboratory of National Forestry and Grassland Administration, Beijing Forestry University, Beijing 100083, China

**Keywords:** *Asparagus officinalis*, NAC transcription factor, gene family, salinity stress, genome-wide analysis, gene duplication, syntenic analysis

## Abstract

As a large plant-specific gene family, the NAC (NAM, ATAF1/2, and CUC2) transcription factor is related to plant growth, development, and response to abiotic stresses. Although the draft genome of garden asparagus (*Asparagus officinalis*) has been released, the genome-wide investigation of the NAC gene family is still unavailable. In this study, a total of 85 *A. officinalis NAC* genes were identified, and a comprehensive analysis of the gene family was performed, including physicochemical properties, phylogenetic relationship, chromosome localization, gene structure, conserved motifs, intron/exon, cis-acting elements, gene duplication, syntenic analysis, and differential gene expression analysis. The phylogenetic analysis demonstrated that there were 14 subgroups in both *A. officinalis* and *Arabidopsis thaliana*, and the genes with a similar gene structure and motif distribution were clustered in the same group. The cis-acting regulatory analysis of *AoNAC* genes indicated four types of cis-acting elements were present in the promoter regions, including light-responsive, hormone-responsive, plant-growth-and-development-related, and stress-responsive elements. The chromosomal localization analysis found that 81 *NAC* genes in *A. officinalis* were unevenly distributed on nine chromosomes, and the gene duplication analysis showed three pairs of tandem duplicated genes and five pairs of segmental duplications, suggesting that gene duplication is possibly associated with the amplification of the *A. officinalis* NAC gene family. The differential gene expression analysis revealed one and three *AoNAC* genes that were upregulated and downregulated under different types of salinity stress, respectively. This study provides insight into the evolution, diversity, and characterization of *NAC* genes in garden asparagus and will be helpful for future understanding of their biological roles and molecular mechanisms in plants.

## 1. Introduction

The transcriptional regulation of gene expression controls many important cellular processes in plants, such as cellular morphogenesis, signal transduction, and environmental stress responses [1]. Transcription factors (TFs) are one kind of regulatory protein that stimulates or inhibits the transcriptional rates of its targeted genes by binding to specific cis-acting elements, thereby controlling plant growth and development, as well as abiotic and biotic stress responses [2,3,4]. According to the different DNA-binding domains (DBDs) in target genes’ promoters, plant transcription factors can be classified into 58 families [5], such as MYB (v-myb avian myeloblastosis viral oncogene homolog), AP2/ERF (APETALA 2/ethylene-responsive element binding factor), HD-Zip (homeodomain leucine zipper), RHR (rel-homology region), Sp1 (specificity protein 1), ARF (auxin response factor), MRKY, NAC, and so on [6].

As one of the significant and diverse plant-specific transcription factor families, the name of the NAC gene family originated from three important proteins with similar DNA-binding domains: NAM (no apical meristem) in *Petunia*, ATAF1/2 (transcription activation factors), and CUC2 (cup-shaped cotyledon 2) in *Arabidopsis* [4,6]. The NAM domain affects the formation and differentiation of apical meristem of *petunia* [6]. ATAF1 and ATAF2 are found to negatively regulate the defense responses against necrotrophic fungal and bacterial pathogens [7]. CUC2 plays an important role in the development of *Arabidopsis* embryos, flowers, and apical meristem [4]. Generally, the N-terminus of NAC proteins contains a well-conserved NAM domain which is helpful to bind the target genes to their cis-acting elements, and the C-terminus contains variable transcriptional activation regions [8]. The N-terminus NAC domain is commonly composed of about 160 amino acids (aa) which can be further cut up into five subdomains, A to E, along with the related functions of nuclear localization, DNA binding, and the construction of homo- and heterodimerize [9,10,11,12,13,14]. The three subdomains A, C, and D are highly conserved; however, the remaining two subdomains, B and E, are poorly conserved, and this may lead to the diversity of NAC protein functions [3]. In contrast, the C-terminus of NAC proteins is highly divergent and has an essential structural basis for the interaction between NAC proteins [15]. Furthermore, the C-terminus of several NAC proteins is membrane-bound, which contributes to rapid transcriptional regulation in endoplasmic reticulum stress response [16].

NAC proteins regulate the growth and development processes of a variety of plants, including the secondary wall formation [17], shoot apical meristem formation [18], organ boundary separation [19], lateral root growth [20], fruit maturation [9], leaf senescence [21,22], and cell-cycle control [23,24]. NAC proteins are also involved in the signal transduction pathways of auxin, cytokinin, ethylene, gibberellin, and other hormones [20,25,26]; for instance, *Arabidopsis ATAF1* can directly regulate the expression of abscisic acid (ABA) synthesis gene NCED3 to regulate ABA biosynthesis [27] and can also induce the expression of defense signal genes related to the jasmonic acid (JA) pathway [28]. Particularly, NAC proteins play an important role in abiotic stresses such as drought, low temperature, high temperature, and high salt; and biological stresses such as pests and pathogens [28,29,30,31,32,33,34,35,36]. One study showed that *OsNAC5* as a rice *NAC* gene enlarged the root diameter through overexpression, so as to enhance drought tolerance and increase grain yield in the field [37]. Another research study revealed that the overexpression of *TaNAC69* in wheat could regulate stress upregulated genes and help wheat adapt to drought stress [38].

In recent years, with the completion of a large number of plant genome sequencings and the continuous improvement of bioinformatics, the research of the NAC transcription factor gene family has entered an upsurge. Up till now, 117 *NAC* genes in *A. thaliana* [39,40], 151 in *Oryza sativa* [39,40,41], 163 in *Populus trichocarpa* [42], 71 in *Cicer arietinum* [43], 83 in *Sesamum indicum* [44], 183 in *Pyrus bretschneideri* [45], 114 in *Betula pendula* [46], and 73 in *Ananas comosus* [47] have been identified by genome-wide analyses, respectively. However, *NAC* genes were not studied in *A. officinalis*.

*A. officinalis* is also known as garden asparagus, whose genome contains 10 chromosomes with a total length of about 1187.54 Mb. Internationally known as “the king of vegetables”, garden asparagus stalk is usually eaten as a vegetable, and it is one of the world’s top ten dishes due to its high nutritional value [48]. Garden asparagus is grown in nearly all areas of the world, with the largest production regions being China, Western Europe, North America, and Peru [49]. Worldwide asparagus production reached 8.45 million metric tons in 2020 [50]. Additionally, its materials have also been used for centuries as herbal medicine because it is rich in amino acids, folic acid, ascorbic acid, phenols, saponins, dietary fiber, anthocyanins, and so on [51]. Soil salinization is one of the main limiting factors of agricultural production which seriously affects the growth and development of plants. It is estimated that about 20% of cultivated land and 33% of irrigated farmland in the world are seriously affected by saline–alkali stress [52]. Garden asparagus is a strong salt-tolerant plant which can grow normally in saline–alkali soil below about 50 mM. It is a pioneer crop for the development and utilization of saline–alkali land and the exploration of plant salt tolerance mechanisms. Therefore, understanding the response of *A. officinalis* to salinity stress is helpful to clarify the mechanism of plant adaptation and obtain a stable yield of plant breeding in saline–alkali soil [53]. Previous studies mainly focused on the analysis of nutritional components and active components in different parts of garden asparagus, whereas there were few studies at the genome level, and the research on its response mechanism to salinity stress is still in its infancy. The garden asparagus genome was completed and published in 2017 [54]; it provides a powerful tool for studying garden asparagus from the gene level. The genome-wide identification and analysis of the NAC gene family will provide an important basis for understanding the evolution of signaling pathways and abiotic stress response, especially salt-stress response. In this research, a comprehensive investigation was conducted of the NAC gene family in garden asparagus, including gene structure, domain analysis, chromosome localization, intron/exon, subcellular localization, and cis-acting elements of *NAC* genes. In addition, we analyzed the phylogenetic relationship of NAC proteins between *A. officinalis* and *A. thaliana*. Furthermore, we investigated the gene duplication pattern of garden asparagus NAC proteins, and we performed a syntenic analysis of NAC proteins among *A. officinalis*, *A. thaliana*, *S. indicum*, and *A. comosus*. Finally, we utilized transcriptome data of garden asparagus to analyze differentially expressed genes under different types of salinity stress. Our study may lay the foundation for a follow-up study of the development, regulation, and biological functions of *NAC* genes in garden asparagus.

## 2. Materials and Methods

### 2.1. Identification of the NAC Gene Family in A. officinalis

Genome sequences, protein sequences, coding sequence (CDS), and annotation files of *A. officinalis* were downloaded from the Ensembl Plants (http://plants.ensembl.org/index.html, accessed on 24 March 2022). The Hidden Markov Model (HMM) file of the NAM domain (PF02365) was retrieved from the Pfam protein family database (release 35.0; http://pfam.xfam.org/, accessed on 24 March 2022) [55].

*A. officinalis* NAC protein sequences were aligned through HMMER search (version 3.1b2) with an e-value of 0.05. To avoid missing candidates, we selected 67 protein sequences with e-value < 1 × 10^−20^ to rebuild a new HMM model, and ClustalW in MEGA 11 software (version 11.0.11) was used for multiple sequence alignment [56,57]. The new HMM model was used to search all *A. officinalis* protein sequences at an e-value of 0.05. We took the intersection of the two search results as the final candidate protein sequences. Furthermore, we utilized the SMART program (http://smart.embl.de/smart/batch.pl, accessed on 26 March 2022) [58], NCBI Conserved Domain Search (https://www.ncbi.nlm.nih.gov/Structure/bwrpsb/bwrpsb.cgi, accessed on 26 March 2022), and Pfam Batch Sequence Search (http://pfam.xfam.org/search#tabview=tab1, accessed on 26 March 2022) to verify the existence of the NAM domain in each candidate protein sequence. After the elimination of the unqualified sequences, 85 *NAC* genes were identified from the *A. officinalis* genome.

### 2.2. Phylogenetic Analysis and Classification of AoNAC Genes

To understand the phylogenetic relationship and to classify the *NAC* genes, the unrooted phylogenetic tree for *A. officinalis* (*AoNAC*) and *A. thaliana* (*AtNAC*) NAC proteins was constructed by using MEGA 11 software (version 11.0.11). The *AoNAC* genes were classified according to their phylogenetic relations, with corresponding *A. thaliana* NAC members. *A. thaliana* NAC protein sequences were obtained from TAIR (https://www.arabidopsis.org, accessed 28 March 2022), with the accession numbers reported by Ooka H et al. [40]. All protein sequences were aligned by Muscle in MEGA 11 software (version 11.0.11), with the default parameters [59]. The Maximum Likelihood (ML) method was used with the following parameters: 1000 iterations for the bootstrap method, Poisson model, and use all sites. Additionally, an individual phylogenetic tree of *AoNAC* genes was built with the same method and visualized by the ggtree package in the R programming language (version 4.1.3) [60].

### 2.3. Chromosomal Mapping and Cis-Acting Regulatory Analysis of AoNAC Genes

The chromosome annotation file of *AoNAC* genes was obtained from Ensembl Plants. The chromosomal position of *AoNAC* genes was displayed by using MG2C (http://mg2c.iask.in/mg2c_v2.1, accessed on 29 March 2022). All identified genes were mapped to 10 chromosomes according to their chromosomal positions and relative distance. The upstream sequences (2000 bp) of *AoNAC* genes’ CDS were retrieved from the *A. officinalis* genome by TBtools software (version 1.098696), according to gene ID, and then submitted to the PlantCARE database (http://bioinformatics.psb.ugent.be/webtools/plantcare/html, accessed on 29 March 2022) to predict cis-acting elements [61]. TBtools software (version 1.098696) was used to visualize the cis-acting elements in upstream sequences after filtering and screening.

### 2.4. Gene Structure and Motif Analysis of AoNAC Genes

The online program MEME (https://meme-suite.org/meme/tools/meme, accessed on 30 March 2022) was applied to analyze the conserved motifs in the *AoNAC* proteins with the following settings: maximum number of motifs 15, minimum motif width 6, maximum motif width 50, and number of repetitions any [62]. The Gene Structure Display Server 2.0 program (http://gsds.gao-lab.org, accessed on 30 March 2022) was used to analyze the intron/exon structure of *AoNAC* genes [63].

### 2.5. Physicochemical Properties and Subcellular Localization Analysis of AoNAC Genes

The ProtParam (https://web.expasy.org/protparam, accessed on 1 April 2022) and Compute pI/Mw (https://web.expasy.org/compute_pi, accessed on 1 April 2022) in the Expasy Webserver were used to analyze physicochemical properties, including the theoretical isoelectric point (pI), molecular weight (MW), instability index, and aliphatic index [64]. For the protein sequences whose theoretical isoelectric point (pI) and molecular weight (MW) could not be predicted by Compute pI/Mw (https://web.expasy.org/compute_pi, accessed on 1 April 2022), DNAman software (version 6.0.3.48) was used for relevant prediction. The number of amino acids (aa) and the open reading frame (ORF) lengths were found on the ORFfinder website (https://www.ncbi.nlm.nih.gov/orffinder, accessed on 1 April 2022). The BUSCA program (https://busca.biocomp.unibo.it, accessed on 1 April 2022) was used to predict the subcellular localization (SL) of the *AoNAC* proteins.

### 2.6. Gene Duplication and Syntenic Analysis of AoNAC Genes

Gene duplications of *AoNAC* genes were predicted through MCScanX in TBtools software (version 1.098696), with default parameters. The duplication events in the *A. officinalis* genome were calculated by Diamond output results in MCScanX. The Duplicate_gene_classifier program in MCScanX (https://github.com/wyp1125/MCScanX, accessed on 3 April 2022) was applied to analyze the duplication type of each *AoNAC* gene. A total of 85 *AoNAC* genes were classified into various types of duplications, including WGD/Segmental, tandem, proximal, singleton, and dispersed. The CDSs of tandem duplicated sequences in *AoNAC* genes were aligned via Muscle (Codons) in MEGA 11 software (version 11.0.11), with the default parameters. The Ka/Ks ratios for tandem duplicated gene pairs in *AoNAC* genes were calculated by using the KaKs_calculator (Version 2.0) with the following settings: genetic code Table 1 (standard code); and method of calculation, YN [65]. The WGD/Segmental duplications were visualized by using the Advanced Circos of TBtools software (version 1.098696). One-Step MCScanX was used to predict the synteny between the *NAC* genes in *A. officinalis* with the *NAC* genes in *A. thaliana*, *A. comosus* (Pineapple), and *S. indicum* (Sesame), using the genome annotation files and genome sequences files. The dual synteny plot for MCScanX in TBtools software (version 1.098696) was used to visualize the synteny.

### 2.7. Differential Expression of AoNAC Genes under Different Types of Salinity Stress

RNA-sequencing data sequenced by Illumina HiSeq 2500 of *A. officinalis* were used to analyze differentially expressed genes of *AoNAC* genes under different types of salinity stress [66]. The experiment consisted of 4 total treatments: (1) non-inoculated *A. officinalis* plants without salinity stress (NI), (2) inoculated *A. officinalis* plants without salinity stress (arbuscular mycorrhiza fungi, AMF), (3) non-inoculated *A. officinalis* plants subjected to salinity stress (NI + S), and (4) inoculated *A. officinalis* plants subjected to salinity stress (AMF + S). Each treatment had 3 biological replicates, and a total of 12 leaf samples were obtained for further analysis. Sequence read archives (SRAs) with accession number SRP188664 were retrieved from the National Centre for Biotechnology Information (NCBI) (https://www.ncbi.nlm.nih.gov/sra/?term=SRP188664#, accessed on 30 April 2022) (Supplementary Table S1). FASTQ files generated the pair-end data containing forward and reverse reads from SRA files. FastQC and MultiQC were used to check the quality of the reads at each step [67,68]. Trimmomatic software was used to trim adapter and low-quality sequences from the reads [69]. The GTF file of garden asparagus was used as the reference genome for the alignment of the reads in STAR [70]. The number of reads per gene was determined by using RSEM software [71]. DESeq2 package was used to analyze differential gene expression of *AoNAC* genes under normal and salt-stress conditions [72]. Filtration of differentially expressed genes was carried out at a significant adjusted *p*-value < 0.05 and an absolute value of log2FC (log of fold change) > 1 to filter out insignificantly expressed genes. The volcano plot was created by using differential expression data from DESeq2. Differentially expressed *AoNAC* genes were labeled in the volcano plot. The heatmap of the differentially expressed genes was constructed by the heatmap package, following the log10 transformed RPKM (the reads per kilobase per million) values.

## 3. Results

### 3.1. Identification of NAC Members in A. officinalis

To identify the *NAC* genes in the *A. officinalis* genome, the Hidden Markov Model (HMM) file corresponding to the NAM domain (PF02365) was employed as a query to search in the *A. officinalis* protein database. After finishing the first HMMER search and removing duplicates, we obtained a total of 86 putative NAC proteins. For the second HMMER search, we selected 67 proteins with an e-value < 10^−20^ as candidate members to rebuild a new HMM model. Based on the results of two HMMER searches, we finally obtained 87 candidate protein sequences. The sequences were further examined by the NCBI Conserved Domain Search, Pfam Batch Sequence Search, and SMART for the authenticity of NAM domains in individual sequences. Finally, the HMMER search and domain analysis identified 85 *NAC* genes in *A. officinalis* (Supplementary Table S2). The identified genes were renamed from *AoNAC5* to *AoNAC85* according to their sequential distribution on chromosomes and subjected to further analyses. The first four *AoNAC* genes (*AoNAC1* to *AoNAC4*) had no annotation information on any *A. officinalis* chromosome, so they were mapped on an undefined chromosome (Un). Detailed characteristics of identified *AoNAC* genes, including the number of amino acids (aa), molecular weight (MW), isoelectric point (pI), instability index, aliphatic index, subcellular localization, and duplication type of *AoNAC* genes are listed in Table 1 and Supplementary Table S3.

The statistical results showed that protein sequences of *AoNAC* genes ranged from 90 amino acid residues (*AoNAC59*) to 1187 amino acid residues (*AoNAC28*), and the molecular weights varied from 10.34 kDa (*AoNAC59*) to 131.61 kDa (*AoNAC28*). The ORFs of the 85 *AoNAC* genes ranged from 273 bp (*AoNAC59*) to 3564 bp (*AoNAC28*). Additionally, 48 *AoNAC* proteins with less than 6.5 pI values were acidic, while 28 with more than 7.5 pI values were alkaline, and 9 with pI values between 6.5 and 7.5 were neutral. Most *AoNAC* proteins had an instability index of more than 40, belonging to unstable proteins, in addition to *AoNAC4*, *AoNAC6*, *AoNAC11*, *AoNAC12*, *AoNAC13*, *AoNAC30*, *AoNAC35*, *AoNAC48*, *AoNAC50*, *AoNAC54*, *AoNAC63*, *AoNAC64*, *AoNAC65*, and *AoNAC80*. The subcellular localization predicted that 89.41% of *AoNAC* genes were located in the nucleus, whereas *AoNAC31*, *AoNAC55*, *AoNAC60*, *AoNAC72*, and *AoNAC78* were found in the chloroplast; *AoNAC14* and *AoNAC22* were found in the endomembrane system; *AoNAC29* was found in the plasma membrane; and *AoNAC69* was found in the extracellular space. The duplication-type analysis of each *AoNAC* gene by MCScanX indicated that most of the genes were dispersed (75.29%) and WGD/Segmental (10.59%), seven of *AoNAC* genes were tandem (8.24%), and five of *AoNAC* genes were proximal (5.88%).

### 3.2. Phylogenetic Analysis of AoNAC and AtNAC

**Figure 1:**
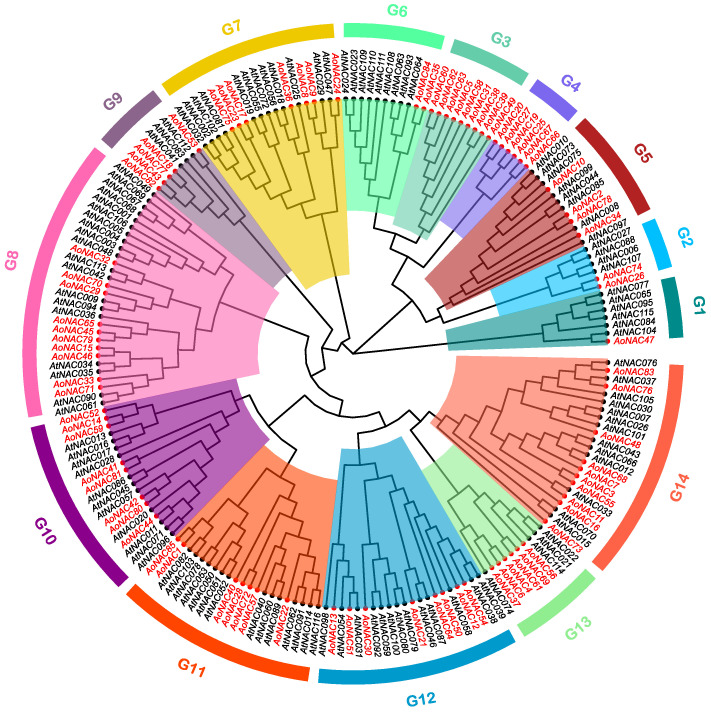
Phylogenetic tree of *NAC* genes between *A. officinalis* and *A. thaliana*. The *AoNAC* and *AtNAC* genes are indicated with red and black fonts, respectively. They are divided into 14 subgroups according to the subgroups of *Arabidopsis* and represented by different colors. The phylogenetic tree was compiled by the Maximum Likelihood (ML) method, with 1000 bootstrap replicates.

To explore the evolutionary relationship of NAC proteins between *A. officinalis* and *A. thaliana*, we constructed an unrooted phylogenetic tree by using MEGA 11 software based on NAC protein full-length sequences alignment of 85 proteins from *A. officinalis* and 116 from *A. thaliana* (Figure 1), and it allowed us to infer the possible functions of *AoNAC* genes based on the function research of *AtNAC* genes. Based on the homology with NAC proteins in *A. thaliana*, the 85 *AoNAC* proteins formed 14 clades (renamed as subgroups G1–G14) together with *AtNAC* proteins. The largest subgroups (G8 and G14) involved ten *AoNAC* members, while the smallest (G1) only had one member. Two subgroups (G3 and G4) only involved the members of *A. officinalis*, meaning that the homologs of *NAC* genes in two subgroups may differentiate during the evolution of *A. thaliana*. It was worth noting that *AtNAC* genes with the same function had a strong tendency to aggregate into the same subgroup. For instance, *CUC1* (*AtNAC*054), *CUC2* (*AtNAC*098), and *CUC3* (*AtNAC*031) involved in shoot organ-boundary separation were mainly located in the subgroup G12. *VND1* (*AtNAC*037), *VND2* (*AtNAC*076), *VND3* (*AtNAC*105), *VND4* (*AtNAC*007), *VND5* (*AtNAC*026), *VND6* (*AtNAC*101), and *VND7* (*AtNAC*030) involved in secondary wall synthesis were mainly located in subgroup G14. The subgroup G7 contained many famous *AtNAC* genes related to stress response, including *AtNAC*019, *AtNAC*056, *AtNAC*055, *AtNAC*002, *AtNAC*081, and *AtNAC*072 (Supplementary Table S4). Therefore, it was speculated that *AoNAC* genes in the corresponding subgroup may have similar functions.

### 3.3. Gene Structure, Conserved Motifs, and Domain Analysis of AoNAC Genes

**Figure 2:**
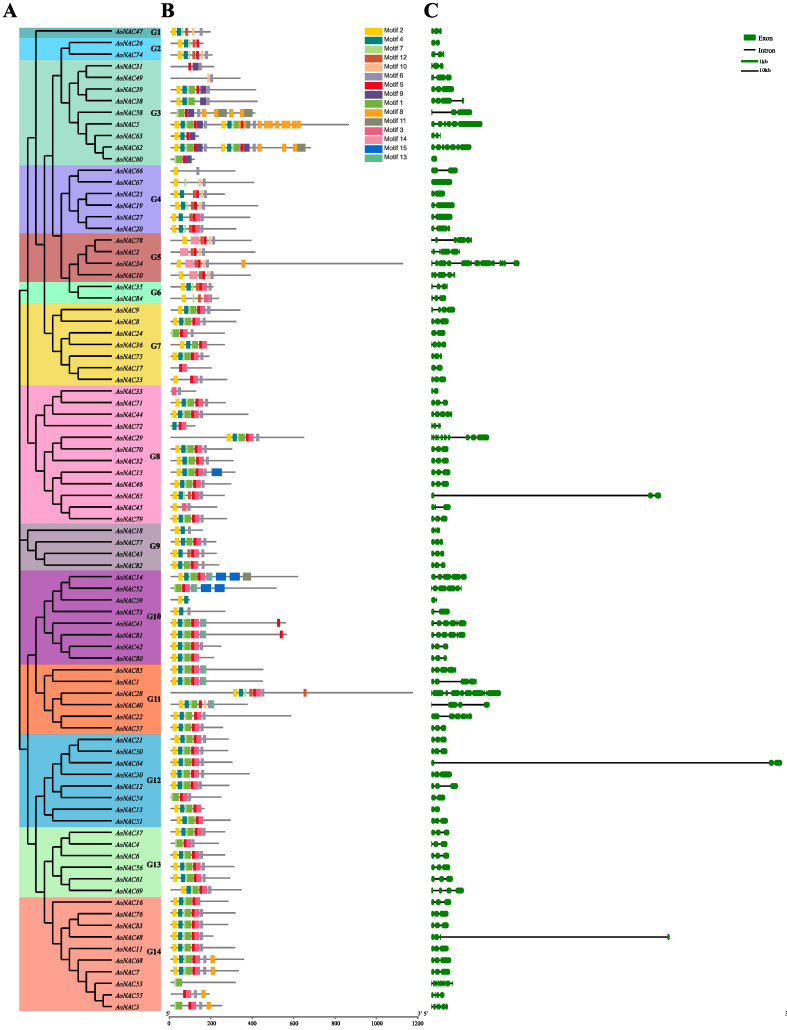
Phylogenetic relationship, conserved motifs, and gene structure of *AoNAC* genes. (**A**) An unrooted phylogenetic tree was constructed by using the ML method with 1000 bootstrap replicates based on *AoNAC* protein full-length sequences. (**B**) The conserved *AoNAC* protein motifs were predicted by the MEME program. Different colored boxes represent different motifs, and the black lines represent non-conserved sequences. The scale bar is 200 amino acids. (**C**) The intron/exon structures of *AoNAC* genes were displayed by using Gene Structure Display Server 2.0 program. The black line represents introns, and the green box represents exons. The intron and exon scale bars are 10 and 1 kb, respectively.

In order to explore the evolutionary relationship between *AoNAC* genes, we established an unrooted phylogenetic tree based on multiple sequence alignment of 85 *AoNAC* proteins. The *AoNAC* genes were separated into 14 subgroups (renamed G1 to G14), and this was basically consistent with the phylogenetic analysis between *AoNAC* and *AtNAC* in the above results (Figure 2A). The largest subgroup (G8) involved 12 members, while the smallest contained 3 members. Moreover, a majority of *AoNAC* genes with similar domains were gathered into a subgroup, such as *AoNAC14*, *AoNAC41*, and *AoNAC81*, with transmembrane domains assigned to the subgroup G10.

In order to further understand the structural diversity and similarity of *AoNAC* genes, we studied the conserved motifs and intron/exon distribution based on their phylogenetic relationship. A total of 15 conservative motifs were predicted by the MEME program and named from Motif 1 to Motif 15 (Figure 2B and Supplementary Table S5). Similar to the domain analysis, *AoNAC* members that were gathered into the same subgroup exhibited a common motif composition, thus implying that their biological functions might be similar. By analyzing the motif distribution of *AoNAC* proteins, the N-terminus of most *AoNAC* genes involved five well-conserved motifs (Motif 2, Motif 4, Motif 5, Motif 6, and Motif 7), which conferred DNA-binding activity. Specific subgroups had different conserved motifs in the C-terminus regions, such as Motif 9 in subgroup G3, Motif 14 in subgroup G5, and Motif 15 in subgroup G10 (Figure 2B), indicating that the specific motifs in different subgroups may bring specific functions.

The intron/exon structure of the *AoNAC* coding sequences was visualized by the Gene Structure Display Server 2.0 program. The result revealed that the number of introns in *AoNAC* genes ranged from zero (*AoNAC60* and *AoNAC67*) to thirteen (*AoNAC34*), and most *AoNAC* genes involved three exons (Figure 2C). Interestingly, *AoNAC* genes in the same phylogenetic group shared highly similar intron/exon structure, differing only in the length of exons and introns.

### 3.4. Chromosomal Mapping and Cis-Acting Regulatory Analysis of AoNAC Genes

**Figure 3:**
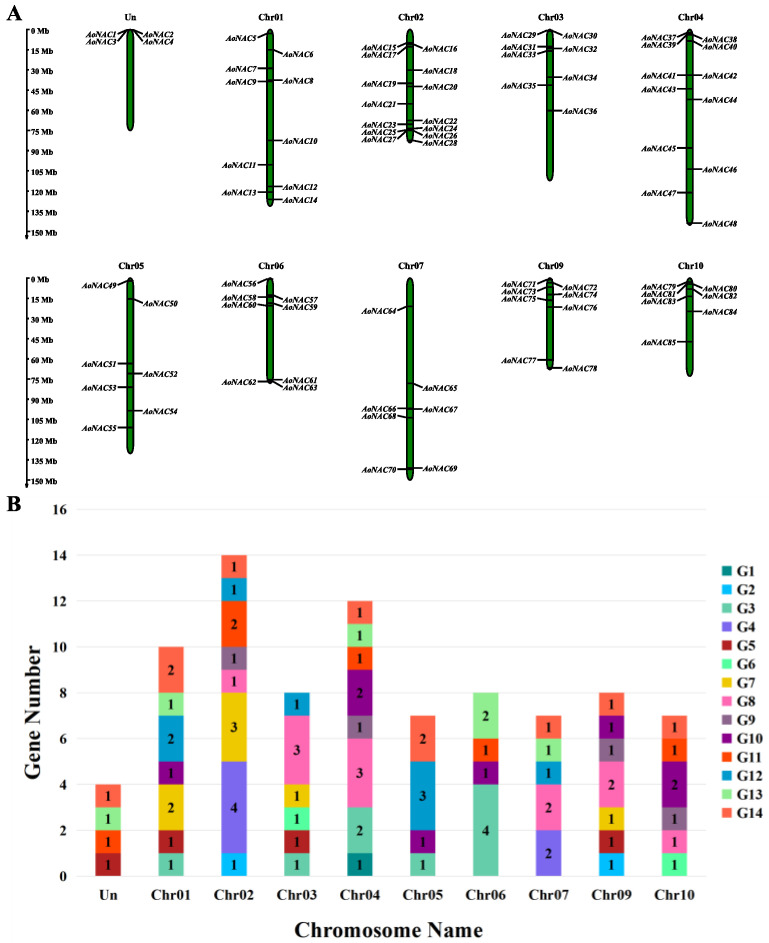
(**A**) Physical distribution of *AoNAC* genes on 9 chromosomes (Chr01–Chr09) and an undefined chromosome (Un). Vertical bars represent the chromosome of *A. officinalis*. The scale is in 15 Mb. (**B**) Number of *AoNAC* subgroups on each chromosome.

We acquired the *AoNAC* genes’ location information according to the genome annotation file from the Ensembl Plants database. Except that chromosome 8 did not contain any *AoNAC* gene, 85 *AoNAC* genes were non-randomly distributed on the remaining nine chromosomes and an undefined chromosome (Un) and were renamed as *AoNAC1*-*AoNAC85* based on their position on the chromosome (Figure 3A). Chr02 contained the largest number (14, 16.47%) of *AoNAC* genes, followed by Chr04 with 12 members (14.12%). In contrast, Chr05, Chr07, and Chr10 contained only seven *AoNAC* genes each (8.24%). As shown in Figure 3B, Chr02 and Chr04 had eight subgroups of *AoNAC* genes, while Chr05 and Chr06 had only four subgroups each. Subgroup G14 was observed on eight chromosomes, except for Chr03 and Chr06, while subgroup G1 was only observed on Chr04.

**Figure 4:**
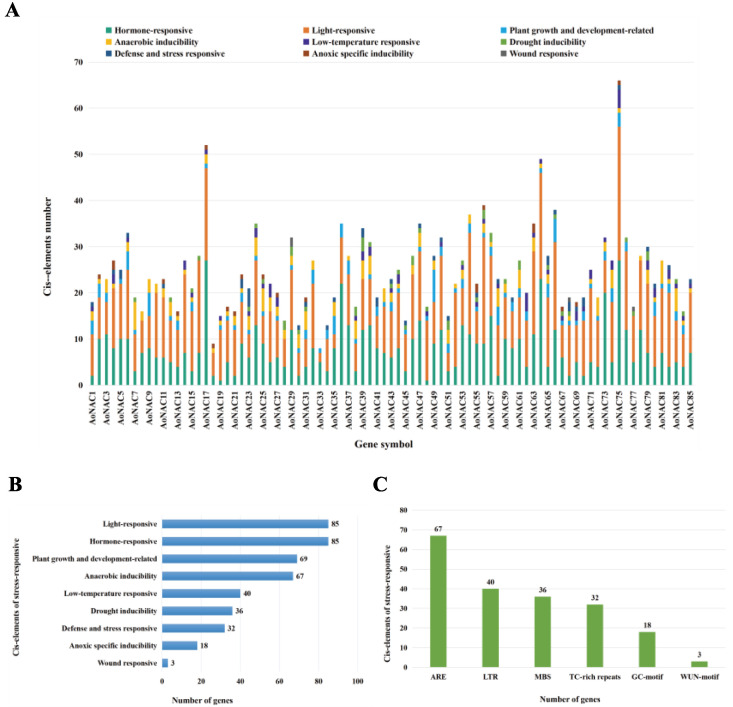
The cis-acting elements analysis of *AoNAC* genes. (**A**) Based on the promoter 2000 bp sequences of 85 *AoNAC* genes, we analyzed the light-responsive, hormone-responsive, plant-growth-and-development-related, anaerobic inducibility, low-temperature responsive, drought inducibility, defense and stress-responsive, anoxic-specific inducibility, and wound-responsive cis-elements. Different colors represent different cis-acting elements. (**B**) The number of *AoNAC* genes in the four types (including six subtypes). (**C**) The number of the various cis-elements in the stress-responsive element is presented in the bar chart.

Cis-acting elements are binding sites of transcription factors, which regulate the precise initiation and efficiency of gene transcription by binding to transcription factors. To deeply study the regulatory mechanism of the *AoNAC* genes in abiotic stress responses, we extracted the 2000 bp sequences upstream of the transcription start site of 85 *AoNAC* genes and then submitted them to the PlantCARE Online program for further analysis. We found four types of cis-acting elements in the promoter regions of the *AoNAC* genes, including light-responsive, hormone-responsive, plant-growth-and-development-related, and stress-responsive elements. The distribution of these cis-acting elements on the promoters is shown in Supplementary Figures S1–S4. The results indicated that *AoNAC* genes were highly relevant to the abiotic stress response. To learn more about the distribution of abiotic stress response, the stress-responsive elements were further divided into anaerobic inducibility, low-temperature responsive, drought inducibility, defense and stress-responsive, anoxic-specific inducibility, and wound-responsive cis-elements (Figure 4A). As shown in Figure 4B, the hormone and light-responsive cis-elements existed in all promoter regions of *AoNAC* genes, the plant-growth-and-development-related cis-elements were present in 69 *AoNAC* genes, the anaerobic inducibility cis-elements were present in 67 *AoNAC* genes, the low-temperature responsive cis-elements were present in 40 *AoNAC* genes, the drought inducibility cis-elements were present in 36 *AoNAC* genes, the defense and stress-responsive cis-elements were present in 32 *AoNAC* genes, the anoxic-specific inducibility cis-elements were present in 18 *AoNAC* genes, and the wound-responsive cis-elements were present in 3 *AoNAC* genes. Furthermore, the stress-responsive elements, including ARE, LTR, MBS, TC-rich repeats, GC-motif, and WUN-motif cis-elements, were related to anaerobic inducibility, low-temperature responsive, drought inducibility, defense and stress-responsive, anoxic specific inducibility, and wound-responsive elements, respectively (Figure 4C). The detailed information about other types of cis-elements in *AoNAC* genes is shown in Supplementary Figures S5 and S6.

### 3.5. Gene Duplication and Syntenic Analysis of AoNAC Genes

**Figure 5:**
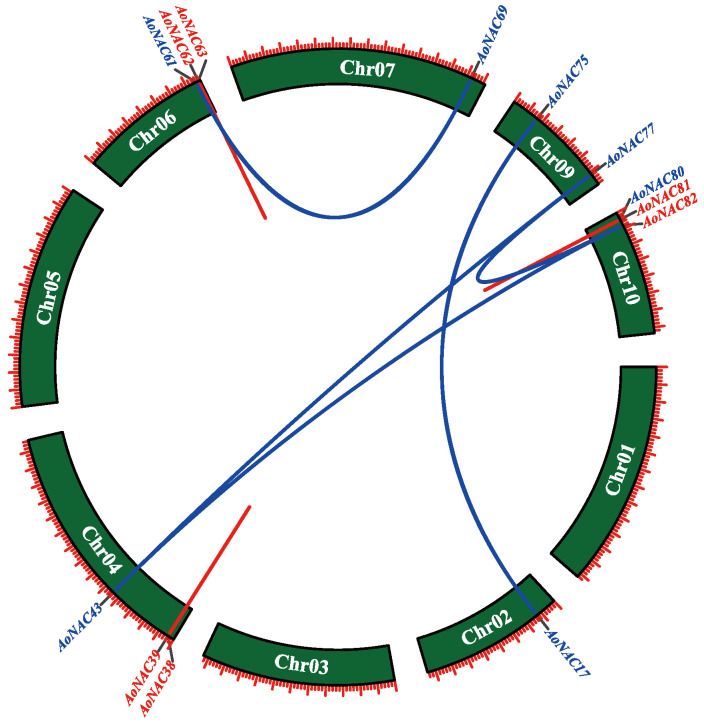
Schematic diagram of the duplication patterns of the *AoNAC* genes. The blue lines indicate segmental duplications of *AoNAC* gene pairs, and the red lines indicate tandem duplications of *AoNAC* gene pairs.

According to the genome-wide analysis of garden asparagus gene duplications generated by MCScanX software, there were 1153 tandem duplications in the genome of *A. officinalis*; however, only three pairs of tandem duplicated genes existed in 81 *AoNAC* genes (Figure 5). The analysis showed that there was one pair of tandem duplicated genes (*AoNAC38* and *AoNAC39*) on Chr04, one pair (*AoNAC62* and *AoNAC63*) on Chr06, and one pair (*AoNAC80* and *AoNAC81*) on Chr10. Additionally, we calculated the substitution ratio of non-synonymous (Ka) to synonymous (Ks) mutations (Ka/Ks) of the above three pairs (Table 2). The Ka/Ks values of the three pairs were less than 1.00, meaning that these duplicated gene pairs evolved under negative purifying selection.

The MCScanX revealed 2336 segmental duplications in the genome of *A. officinalis*; however, only five pairs of segmental duplicated genes were predicted in 81 *AoNAC* genes. The synteny regions (segmental duplications) on all 9 chromosomes were visualized by using TBtools software (Version 1.098696), as represented in Figure 5. Chr09 contained two segmental duplicated genes, while Chr04, Chr06, Chr07, and Chr10 each contained only one segmental duplicated gene. However, Chr01, Chr03, and Chr05 did not contain any segmental duplicated genes.

**Figure 6:**
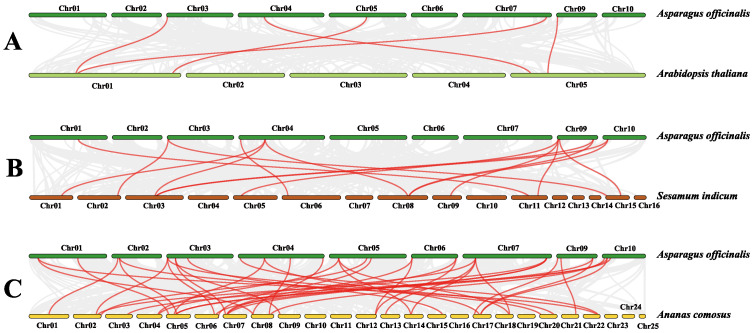
Schematic diagram of syntenic analysis. Synteny of the *AoNAC* genes with the *NAC* genes of *A. thaliana* (**A**), *S. indicum* (**B**), and *A. comosus* (**C**) was visualized by MCScanX and TBtools software. The gray lines between the chromosomes of the two species indicate all synteny blocks, and the red lines represent the synteny of their *NAC* genes.

**Figure 7:**
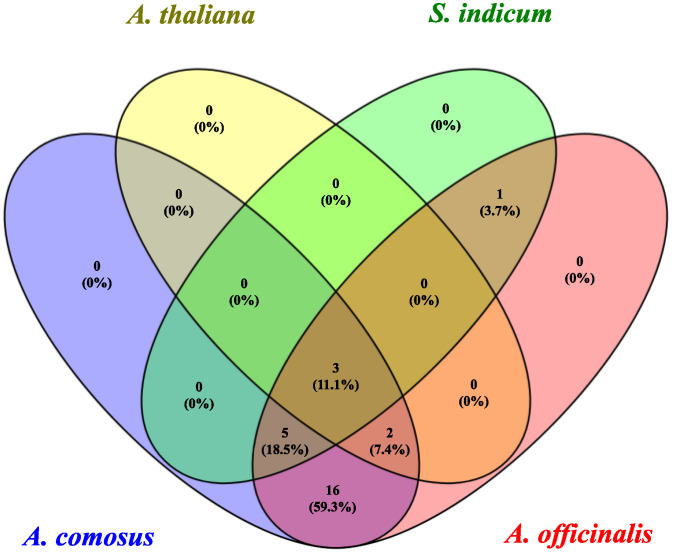
Venn diagram of the identical and different *NAC* genes among *A. officinalis*, *A. thaliana*, *S. indicum*, and *A. comosus*.

The *AoNAC* gene and the *NAC* genes of *A. thaliana*, *S. indicum*, and *A. comosus* were separately analyzed to find homologous gene pairs (Figure 6). We found that 27 *AoNAC* genes were syntenic with the *NAC* genes of *A. thaliana* (4), *S. indicum* (11), and *A. comosus* (27) (Supplementary Tables S6–S8). Considering that several genes had multicollinearity with *NAC* genes of other species, we found that there were 5, 15, and 41 *NAC* genes of *A. thaliana*, *S. indicum*, and *A. comosus*, respectively, which had synteny with 27 *AoNAC* genes. In addition, three *NAC* genes existed in four plants at the same time (Figure 7). However, sixteen homologous *NAC* genes existed in *A. officinalis* and *A. comosus* rather than in *A. thaliana* and *S. indicum*. Similarly, one homologous NAC gene existed in *A. officinalis* and *S. indicum* rather than in *A. thaliana* and *A. comosus*. *A. officinalis*, *A. comosus*, and *S. indicum* had five homologous *NAC* genes that did not exist in *A. thaliana.* Moreover, *A. officinalis*, *A. comosus*, and *A. thaliana* had two homologous *NAC* genes that did not exist in *S. indicum*.

### 3.6. Differentially Expressed Genes Analysis of AoNAC Genes under Different Types of Salinity Stress

**Figure 8:**
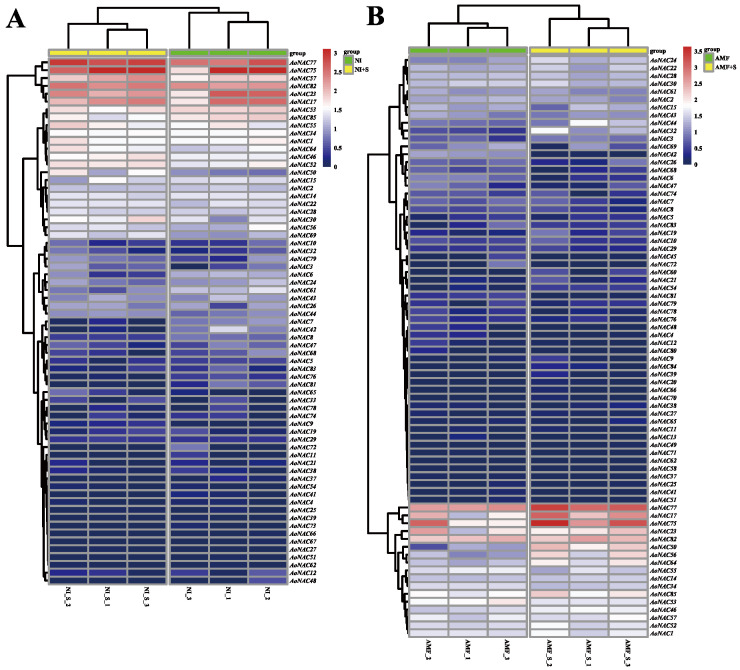
Differential gene expression of *AoNAC* genes under different types of salinity stress: (**A**) non-inoculated *A. officinalis* plants without salinity stress (NI) and non-inoculated *A. officinalis* plants subjected to salinity stress (NI + S); (**B**) inoculated *A. officinalis* plants without salinity stress (AMF) and inoculated *A. officinalis* plants subjected to salinity stress (AMF + S). Heatmaps are based on the log10-transformed RPKM values. Red represents a high expression level, and blue represents a low expression level. Volcano plots are based on the significantly adjusted *p*-value (padj) < 0.05 and an absolute value of log2FC (log of fold change) > 1.

To further provide information on the function of *AoNAC* genes in garden asparagus, we investigated the gene expression difference in *AoNAC* genes under different types of salinity stress, using RNA-seq data. A total of 67 *AoNAC* genes common to NI and NI + S plants were expressed differentially after only salinity treatment (Figure 8A). Among these widely expressed *AoNAC* genes, seven genes were highly expressed under NI and NI + S conditions, especially *AoNAC*75 and *AoNAC*77. In the first 15 *AoNAC* genes in Figure 8A, the average expression of 11 *AoNAC* genes in NI + S treatment was higher than that in NI treatment. In these 67 *AoNAC* genes, 5 (*AoNAC*30, *AoNAC*50, *AoNAC*57, *AoNAC*69, and *AoNAC*77) were significantly upregulated (padj < 0.05 and log2FC > 1) and 5 (*AoNAC*6, *AoNAC*7, *AoNAC*42, *AoNAC*61, and *AoNAC*81) were downregulated (padj < 0.05 and log2FC < −1) in leaves of both NI and NI + S plants during salinity treatment (Supplementary Figure S7A). A total of 74 *AoNAC* genes common to AMF and AMF + S plants were expressed differentially after AMF and salinity treatment (Figure 8B). Among these widely expressed *AoNAC* genes, five genes showed relatively higher expression levels under AMF and AMF + S conditions, but, obviously, the expression of *AoNAC*77, *AoNAC*17, and *AoNAC*75 genes in the AMF plant was much higher than that in the AMF + S plant. In the first 17 *AoNAC* genes shown in Figure 8A, the average expression of 14 *AoNAC* genes in the AMF + S treatment was higher than that in the AMF treatment. In these 74 *AoNAC* genes, 7 (*AoNAC*17, *AoNAC*24, *AoNAC*32, *AoNAC*44, *AoNAC*50, *AoNAC*56, and *AoNAC*77) were significantly upregulated and 3 (*AoNAC*6, *AoNAC*42, and *AoNAC*81) were downregulated in the leaves of both AMF and AMF + S plants during AMF and salinity treatment (Supplementary Figure S7B).

## 4. Discussion

*A. officinalis*, a species belonging to the *Liliaceae* family, is a perennial herb with important economic and pharmacological value. It contains about 300 species with known traditional uses, such as appetizer, lactating enhancer, antioxidant activities, and antitumor [73], while it is also sensitive to stress, such as drought and salinity, as most horticultural crops are [74]. Researchers have identified the NAC gene family in many species; however, little is known about the family in garden asparagus. With the release of the *A. officinalis* genome, transcriptome sequencing and functional genomics have greatly facilitated *A. officinalis* research [75,76]. The NAC gene family is one of the largest families of transcription factors and plays important roles in plant growth, development, and response to abiotic and biotic stresses. In this study, 85 *NAC* genes were identified in the garden asparagus genome, which is less than those of *A. thaliana* (117 *NAC* genes) and similar to those of *S. indicum* (83 *NAC* genes) [40,44]. However, it is more than the number of NAC genes in *A. comosus* (73 *NAC* genes) [47], thereby indicating that more *NAC* genes were needed in the transcriptional regulation of garden asparagus. These results indicated that most *AoNAC* genes were not eliminated by environmental selection; instead, they showed high conservation during the evolution process, which needs to be deeply studied from an evolutionary perspective. These 85 *AoNAC* genes were classified into 14 subgroups according to their phylogenetic relationship with *A. thaliana*. We found that the NAC gene family members were unevenly distributed among subgroups; for instance, the subgroups of G3 and G4 only contained *A. officinalis* members, and the member number of subgroups of G9 and G13 in *A. officinalis* was more than that in *A. thaliana.* Since *A. officinalis* and *A. thaliana* were exposed to different environments during the evolution process, the number of *NAC* genes in their subgroups became different as *NAC* genes differentiated. Gene duplication is closely related to the evolution of genome size, the origin of new genes, species differentiation, and the ability of gene anti-mutation [51,77]. The collinearity analysis in our study showed that there were three pairs of tandem duplication and five pairs of segmental duplication events in the *A. officinalis* NAC gene family, and this might play an important role in the NAC family expansion in garden asparagus.

During the development and evolution of the gene family, the gene structure will vary according to the environmental changes to obtain new functions. The structural analysis of *AoNAC* genes according to phylogenetic relationship revealed that different subgroups have different gene structures and conserved motifs, while the identical subgroup had similar motif compositions and gene structures, thus implying that the members in the same subgroup possessed similar functions. These results were in agreement with reports in *A. thaliana*, *O. sativa*, and *Vitis vinifera* [40,78], which also indicated that NAC proteins with similar structures and motifs within species were functionally orthologous. In this study, we found numerous cis-acting elements that were involved in light-responsive, hormone-responsive, plant growth and development-related and stress-responsive elements. In the hormone-responsive elements as shown in Supplementary Figure S5, ABRE involved in ABA responsiveness existed in 66 *AoNAC* genes, which contributes to regulating strawberry fruit ripening by ABA [14,79]; both CGTCA-motif and TGACG-motif involved in MeJA responsiveness were found in 63 *AoNAC* genes, which activates MYB to regulate overexpression of *MdMYB9* or *MdMYB11* so as to anthocyanin and proanthocyanidin accumulation in apple calluses [80]; previous studies showed that the interaction of light, ethylene, and auxin can regulate the biosynthesis of carotenoids during tomato fruit ripening [81], and TGA-element, AuxRR-core, and TGA-box, involved in auxin-responsive elements, were found in 36, 11, and 1 *AoNAC* genes, respectively; P-box, GARE-motif, and TATC-box, involved in gibberellin-responsive elements, were found in 28, 19, and 18 *AoNAC* genes, respectively; SARE and TCA-element involved in salicylic acid-responsive elements were found in 2 and 30 *AoNAC* genes, respectively. In the plant growth and development-related elements as shown in Supplementary Figure S6, O2-site (zein metabolism regulation), MBSI (MYB binding site involved in flavonoid biosynthetic genes regulation), MSA-like (cell cycle regulation), HD-Zip 1 (differentiation of the palisade mesophyll cells), and HD-Zip 3 (protein binding site), involved in metabolism-related element, were found in 32, 5, 3, 3, and 1 *AoNAC* genes, respectively; CAT-box related to meristem expression was found in 27 *AoNAC* genes; circadian involved in circadian control was found in 16 *AoNAC* genes; RY-element involved in seed-specific regulation was found in 9 *AoNAC* genes; AACA_motif and GCN4_motif involved in endosperm-related element were found in 2 and 14 *AoNAC* genes, respectively.

RNA-seq technology is an indispensable tool for analyzing differential gene expression at the transcriptome level. In this study, transcriptome data of garden asparagus under different types of salinity stress were used to determine the expression of *AoNAC* genes. Whether NI + S treatment or AMF + S treatment, *AoNAC*77 had up-regulated expression whereas *AoNAC*6, *AoNAC*42, and *AoNAC*81 had down-regulated expression. *AoNAC*77 and *AoNAC*75 were the highest expression genes under the four treatments, which means that they may be salt stress tolerance-related genes. Similar to *A. officinalis*, it has been found that *VvNAC17* in *V. vinifera* could enhance salinity, freezing, and drought tolerance in transgenic *Arabidopsis* [82], *GmNAC06* in *Glycine max* played a role in response to salt stress thought controlling the Na+/K+ ratios in hairy roots to maintain ionic homeostasis [83], and *MdNAC047* in apple could enhance salt stress tolerance by modulating the ethylene response [84]. These findings may help to lay foundations for subsequent in-depth research of the specific functions of NAC transcription factor family genes of *A. officinalis*.

## 5. Conclusions

Using a genome-wide identification and analysis of the *A. officinalis* NAC transcription factor family, we identified a total of 85 *AoNAC* genes belonging to fourteen subgroups that were non-randomly distributed across nine chromosomes and an undefined chromosome. Moreover, these proteins had typical NAC-conserved motifs and gene structures within the same subgroup, and they may be involved in light-responsive, hormone-responsive, plant-growth-and-development-related, and stress-responsive elements. Furthermore, segmental duplications in *AoNAC* genes contribute significantly to the expansion of the garden asparagus NAC gene family, and their differential gene expression was significantly influenced by different types of salinity stress. In short, our findings provide more information about *NAC* genes and establish a foundation for future study of *NAC* genes in garden asparagus.

## Figures and Tables

**Table 1:** Detailed information of *NAC* genes in *A. officinalis*.

Gene Symbol	pI	MW (kDa)	Length (aa)	Instability Index	Aliphatic Index	Subcellular Localization	ORF	Duplications
*AoNAC1*	4.49	50.35	449	45.65	63.63	Nucleus	1350	Dispersed
*AoNAC2*	5.18	46.29	412	48.10	70.49	Nucleus	1239	Dispersed
*AoNAC3*	7.68	28.42	249	40.89	70.12	Nucleus	750	Dispersed
*AoNAC4*	5.51	26.46	232	24.75	57.11	Nucleus	699	Dispersed
*AoNAC5*	5.42	99.26	872	57.24	71.69	Nucleus	2619	Dispersed
*AoNAC6*	5.38	29.65	263	39.13	60.72	Nucleus	792	Dispersed
*AoNAC7*	7.38	36.74	330	44.47	65.00	Nucleus	993	Dispersed
*AoNAC8*	8.36	35.47	318	43.20	61.32	Nucleus	957	Dispersed
*AoNAC9*	7.03	37.60	338	44.81	69.56	Nucleus	1017	Dispersed
*AoNAC10*	7.35	43.48	389	47.84	61.13	Nucleus	1167	Dispersed
*AoNAC11*	7.71	35.90	312	39.64	63.46	Nucleus	939	Dispersed
*AoNAC12*	8.54	32.34	284	30.05	58.31	Nucleus	855	Dispersed
*AoNAC13*	9.33	18.93	161	35.46	55.78	Nucleus	486	Dispersed
*AoNAC14*	4.82	69.33	621	54.87	69.73	Endomembrane System	1866	Dispersed
*AoNAC15*	5.95	35.97	313	57.04	68.18	Nucleus	942	Dispersed
*AoNAC16*	6.36	31.38	279	41.39	55.63	Nucleus	840	Dispersed
*AoNAC17*	9.17	21.54	197	61.85	60.46	Nucleus	594	WGD/Segmental
*AoNAC18*	5.49	17.86	153	72.43	74.51	Nucleus	462	Dispersed
*AoNAC19*	4.94	48.28	425	60.37	64.05	Nucleus	1278	Dispersed
*AoNAC20*	5.30	36.19	318	44.07	64.69	Nucleus	957	Dispersed
*AoNAC21*	6.47	31.90	280	44.93	58.57	Nucleus	843	Dispersed
*AoNAC22*	4.84	66.29	589	53.84	69.56	Endomembrane System	1770	Dispersed
*AoNAC23*	6.91	30.50	273	56.59	57.58	Nucleus	822	Dispersed
*AoNAC24*	8.94	30.04	261	45.37	62.03	Nucleus	786	Dispersed
*AoNAC25*	5.81	29.51	261	61.51	68.70	Nucleus	786	Dispersed
*AoNAC26*	5.18	17.86	156	55.88	76.79	Nucleus	471	Dispersed
*AoNAC27*	5.43	43.32	386	54.38	63.73	Nucleus	1161	Proximal
*AoNAC28*	6.44	131.61	1187	53.51	70.73	Nucleus	3564	Tandem
*AoNAC29*	5.93	72.81	652	46.37	78.70	Plasma Membrane	1959	WGD/Segmental
*AoNAC30*	6.47	42.88	383	35.15	60.34	Nucleus	1152	Dispersed
*AoNAC31*	9.73	23.79	209	45.91	56.08	Chloroplast	630	Dispersed
*AoNAC32*	5.76	35.25	304	42.87	59.61	Nucleus	915	Dispersed
*AoNAC33*	4.67	13.56	121	46.42	43.55	Nucleus	366	Dispersed
*AoNAC34*	5.84	127.90	1139	58.08	74.12	Nucleus	3420	Dispersed
*AoNAC35*	9.08	23.61	205	35.37	70.83	Nucleus	618	Dispersed
*AoNAC36*	10.05	27.51	261	40.66	59.20	Nucleus	786	Dispersed
*AoNAC37*	5.78	29.86	263	54.69	61.86	Nucleus	792	Dispersed
*AoNAC38*	5.28	48.40	423	49.84	62.53	Nucleus	1272	Tandem
*AoNAC39*	6.49	47.18	415	53.29	57.42	Nucleus	1248	Tandem
*AoNAC40*	4.52	42.57	373	49.98	66.89	Nucleus	1122	Dispersed
*AoNAC41*	5.71	64.44	563	46.69	73.53	Nucleus	1692	Proximal
*AoNAC42*	4.93	27.97	243	41.99	62.55	Nucleus	732	Proximal
*AoNAC43*	9.02	25.18	221	44.43	56.88	Nucleus	666	WGD/Segmental
*AoNAC44*	5.04	42.46	377	47.30	60.24	Nucleus	1134	Dispersed
*AoNAC45*	4.70	25.48	224	49.37	77.50	Nucleus	675	Dispersed
*AoNAC46*	7.07	33.31	293	56.01	60.31	Nucleus	882	Dispersed
*AoNAC47*	4.91	21.99	191	47.58	63.30	Nucleus	576	Dispersed
*AoNAC48*	9.11	24.52	205	30.90	58.44	Nucleus	618	Dispersed
*AoNAC49*	4.83	37.71	338	67.48	69.56	Nucleus	1017	Dispersed
*AoNAC50*	7.02	32.15	278	33.72	56.47	Nucleus	837	Dispersed
*AoNAC51*	5.03	32.27	290	41.81	74.38	Nucleus	873	Dispersed
*AoNAC52*	4.67	57.78	516	43.74	60.31	Nucleus	1551	Dispersed
*AoNAC53*	9.10	36.03	315	40.01	78.03	Nucleus	948	Dispersed
*AoNAC54*	9.44	27.50	245	38.85	61.76	Nucleus	738	Dispersed
*AoNAC55*	9.46	20.95	186	48.11	81.24	Chloroplast	561	Dispersed
*AoNAC56*	8.04	34.76	308	47.17	61.43	Nucleus	927	Dispersed
*AoNAC57*	5.85	28.97	253	52.15	68.58	Nucleus	762	Dispersed
*AoNAC58*	8.84	46.54	412	53.06	59.13	Nucleus	1239	Dispersed
*AoNAC59*	5.87	10.34	90	48.36	75.78	Nucleus	273	Dispersed
*AoNAC60*	9.27	13.36	113	46.58	48.32	Chloroplast	342	Dispersed
*AoNAC61*	8.44	32.25	287	51.53	58.75	Nucleus	864	WGD/Segmental
*AoNAC62*	6.36	78.39	685	53.96	64.89	Nucleus	2058	Tandem
*AoNAC63*	5.44	15.78	134	39.32	90.90	Nucleus	405	Tandem
*AoNAC64*	7.61	34.01	299	29.06	52.81	Nucleus	900	Dispersed
*AoNAC65*	6.98	29.38	260	38.02	70.50	Nucleus	783	Dispersed
*AoNAC66*	5.47	35.20	313	57.92	61.79	Nucleus	942	Proximal
*AoNAC67*	4.67	45.48	404	57.87	69.33	Nucleus	1215	Proximal
*AoNAC68*	6.67	40.15	356	44.86	71.24	Nucleus	1071	Dispersed
*AoNAC69*	7.04	39.23	344	46.40	57.50	Extracellular Space	1035	WGD/Segmental
*AoNAC70*	7.71	34.28	298	49.56	59.30	Nucleus	897	WGD/Segmental
*AoNAC71*	6.11	29.64	266	65.67	48.08	Nucleus	801	Dispersed
*AoNAC72*	8.88	13.70	117	71.07	64.10	Chloroplast	354	Dispersed
*AoNAC73*	5.50	29.51	264	52.09	62.05	Nucleus	795	Dispersed
*AoNAC74*	8.39	23.07	201	48.54	65.87	Nucleus	606	Dispersed
*AoNAC75*	9.71	20.87	186	44.04	66.18	Nucleus	561	WGD/Segmental
*AoNAC76*	5.51	36.10	314	43.35	61.82	Nucleus	945	Dispersed
*AoNAC77*	9.53	24.87	219	46.45	59.22	Nucleus	660	WGD/Segmental
*AoNAC78*	5.61	44.10	392	42.41	76.12	Chloroplast	1179	Dispersed
*AoNAC79*	8.85	30.95	272	46.24	76.69	Nucleus	819	Dispersed
*AoNAC80*	5.43	24.00	208	36.83	53.41	Nucleus	627	Tandem
*AoNAC81*	5.24	64.63	567	46.76	71.55	Nucleus	1704	Tandem
*AoNAC82*	8.55	26.80	234	57.68	67.91	Nucleus	705	WGD/Segmental
*AoNAC83*	5.58	32.47	278	49.19	64.89	Nucleus	837	Dispersed
*AoNAC84*	8.19	27.05	232	49.19	68.10	Nucleus	699	Dispersed
*AoNAC85*	4.63	50.48	451	40.36	62.86	Nucleus	1356	Dispersed

**Table 2:** Tandem duplication in *AoNAC* genes and corresponding Ka, Ks, and Ka/Ks values.

Tandem Duplication	Chromosome Name	Ka	Ks	Ka/Ks
*AoNAC38* and *AoNAC39*	Chr04	0.35	0.70	0.51
*AoNAC62* and *AoNAC63*	Chr06	0.06	0.10	0.60
*AoNAC80* and *AoNAC81*	Chr10	0.29	3.69	0.08

## Data Availability

Data are available upon reasonable request.

## Supplementary Materials

The following supporting information can be downloaded at https://www.mdpi.com/article/10.3390/genes13060976/s1. Table S1: SRA accession number about *Asparagus officinalis* transcriptome data. Table S2: The NAC protein full-length sequences of *Asparagus officinalis* identified in this study. Table S3: Detailed information of NAC genes in *Asparagus officinalis*. Table S4: The accession numbers of *NAC* genes in *Arabidopsis thaliana*. Table S5: Sequence logos for the conserved motifs of *Asparagus officinalis* NAC domain proteins. Table S6: One-to-one orthologous relationship of *NAC* genes of *Asparagus Officinalis* and *Arabidopsis thaliana*. Table S7: One-to-one orthologous relationship of *NAC* genes of *Asparagus Officinalis* and *Sesamum indicum*. Table S8: One-to-one orthologous relationship of *NAC* genes of *Asparagus Officinalis* and *Ananas comosus*. Figure S1: Light-responsive element in 85 *AoNAC* genes. Figure S2: Plant-growth-and-development-related element in 69 *AoNAC* genes. Figure S3: Hormone-responsive element in 85 *AoNAC* genes. Figure S4: Stress-responsive element in 83 *AoNAC* genes. Figure S5: The analysis of plant-growth-and-development-related element in 69 *AoNAC* genes. Figure S6: The analysis of hormone-responsive element in 85 *AoNAC* genes. Figure S7: Volcanic maps of differential gene expression of *AoNAC* genes under different types of salinity stress.
