# Supplementary material for: Genome-Wide Identification and Analysis of the NAC Transcription Factor Gene Family in Garden Asparagus (Asparagus officinalis)
Source: Genes (Basel). 2022 May 30;13(6):976. doi: 10.3390/genes13060976 (PMC9222252; doi:10.3390/genes13060976)
Supplement: Supplementary file 1 [file genes-13-00976-s001.zip › Supplementary Files/Figure S6-The analysis of hormone-responsive element in 85 AoNAC genes.pdf]

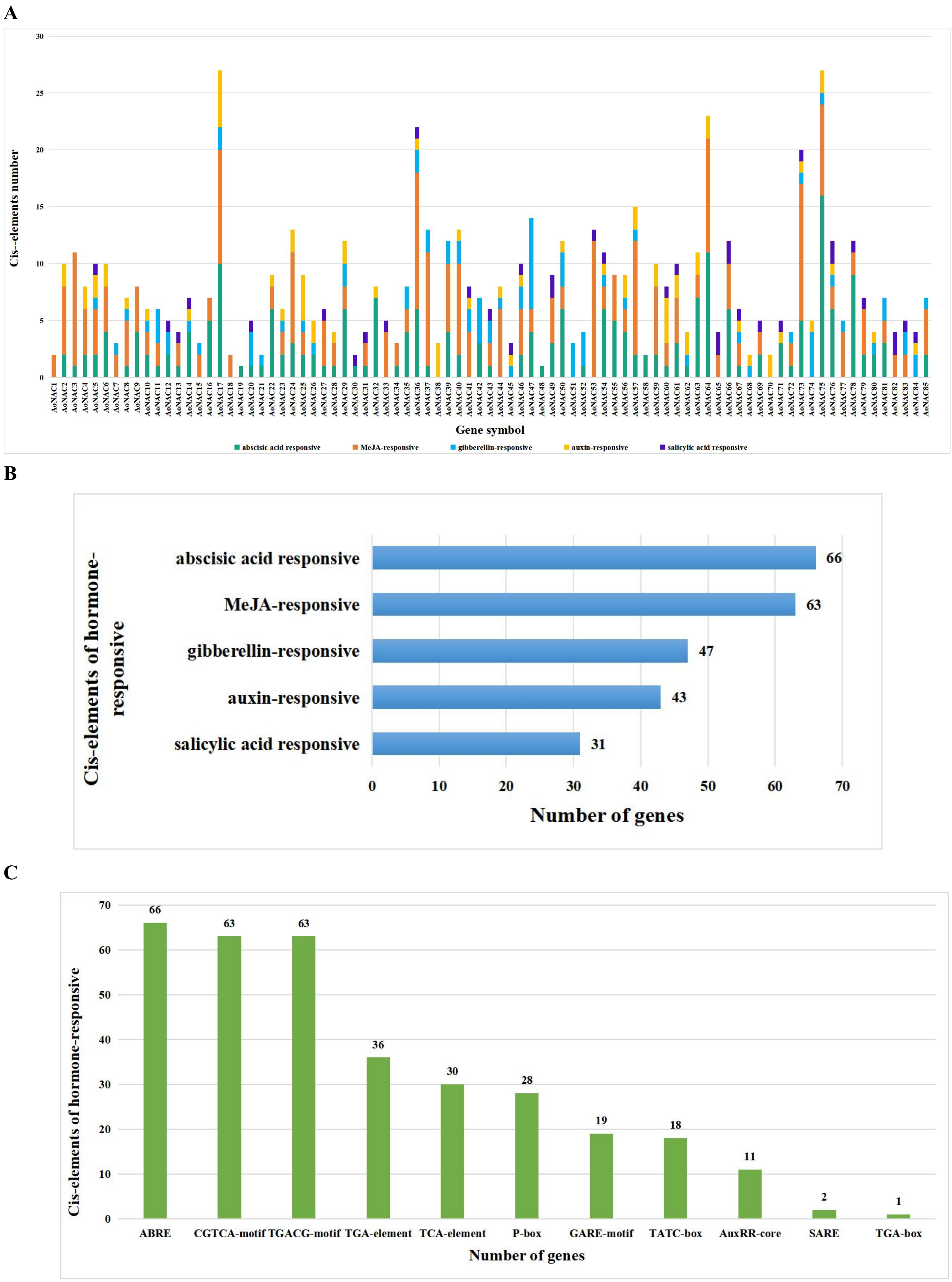

Figure S6. The analysis of hormone-responsive element in 85 *AoNAC* genes. (**A**) Detailed information regarding hormone-responsive element in 85 *AoNAC* genes. (**B**) The number of *AoNAC* genes in 5 subtypes. (**C**) The number of the various cis-elements in the hormone-responsive element.
