# Supplementary material for: Genome-Wide Identification and Analysis of the NAC Transcription Factor Gene Family in Garden Asparagus (Asparagus officinalis)
Source: Genes (Basel). 2022 May 30;13(6):976. doi: 10.3390/genes13060976 (PMC9222252; doi:10.3390/genes13060976)
Supplement: Supplementary file 1 [file genes-13-00976-s001.zip › Supplementary Files/Figure S7-Volcanic maps of differential gene expression of AoNAC genes under different types of salinity stress.pdf]

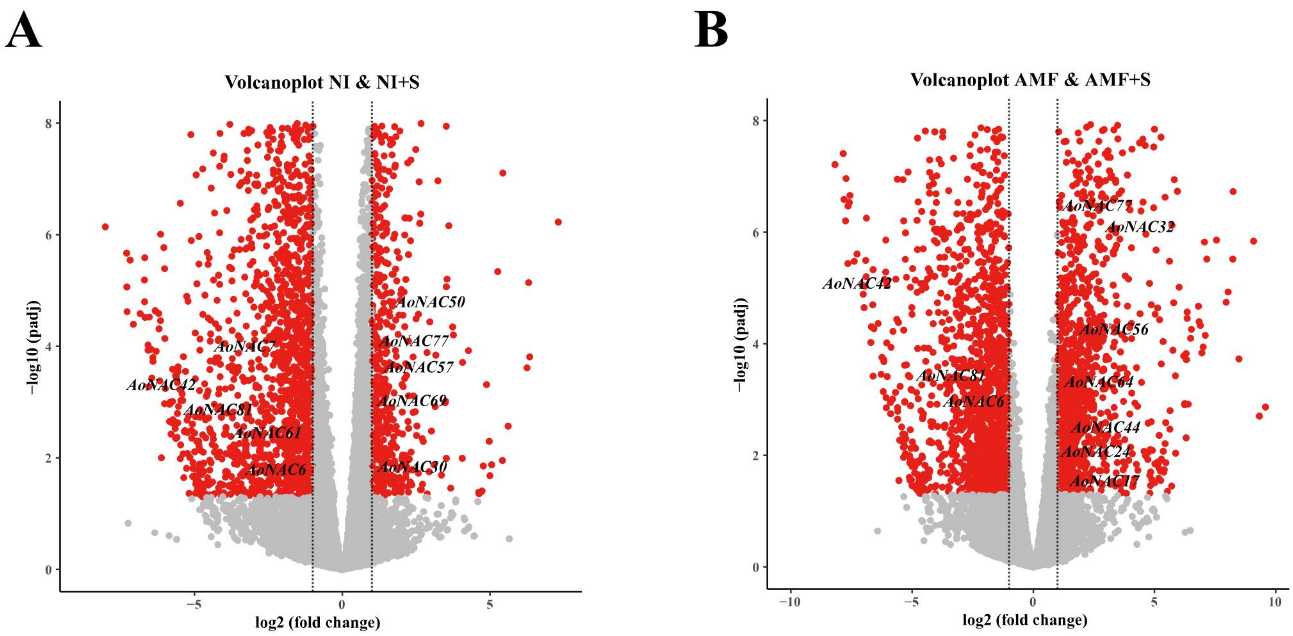

Figure S7. Volcanic maps of differential gene expression of *AoNAC* genes under different types of salinity stress. (**A**) non-inoculated *A. officinalis* plants without salinity stress (NI) and non-inoculated *A. officinalis* plants subjected to salinity stress (NI + S); (**B**) inoculated A. officinalis plants without salinity stress (AMF) and inoculated *A. officinalis* plants subjected to salinity stress (AMF + S). Volcano plots are based on the significant adjusted p-value (padj) < 0.05 and an absolute value of log2FC (log of fold change) > 1.
