# Supplementary figures and images for: Genome-Wide Identification and Analysis of the NAC Transcription Factor Gene Family in Garden Asparagus (Asparagus officinalis)
Source: Genes (Basel). 2022 May 30;13(6):976. doi: 10.3390/genes13060976 (PMC9222252; doi:10.3390/genes13060976)

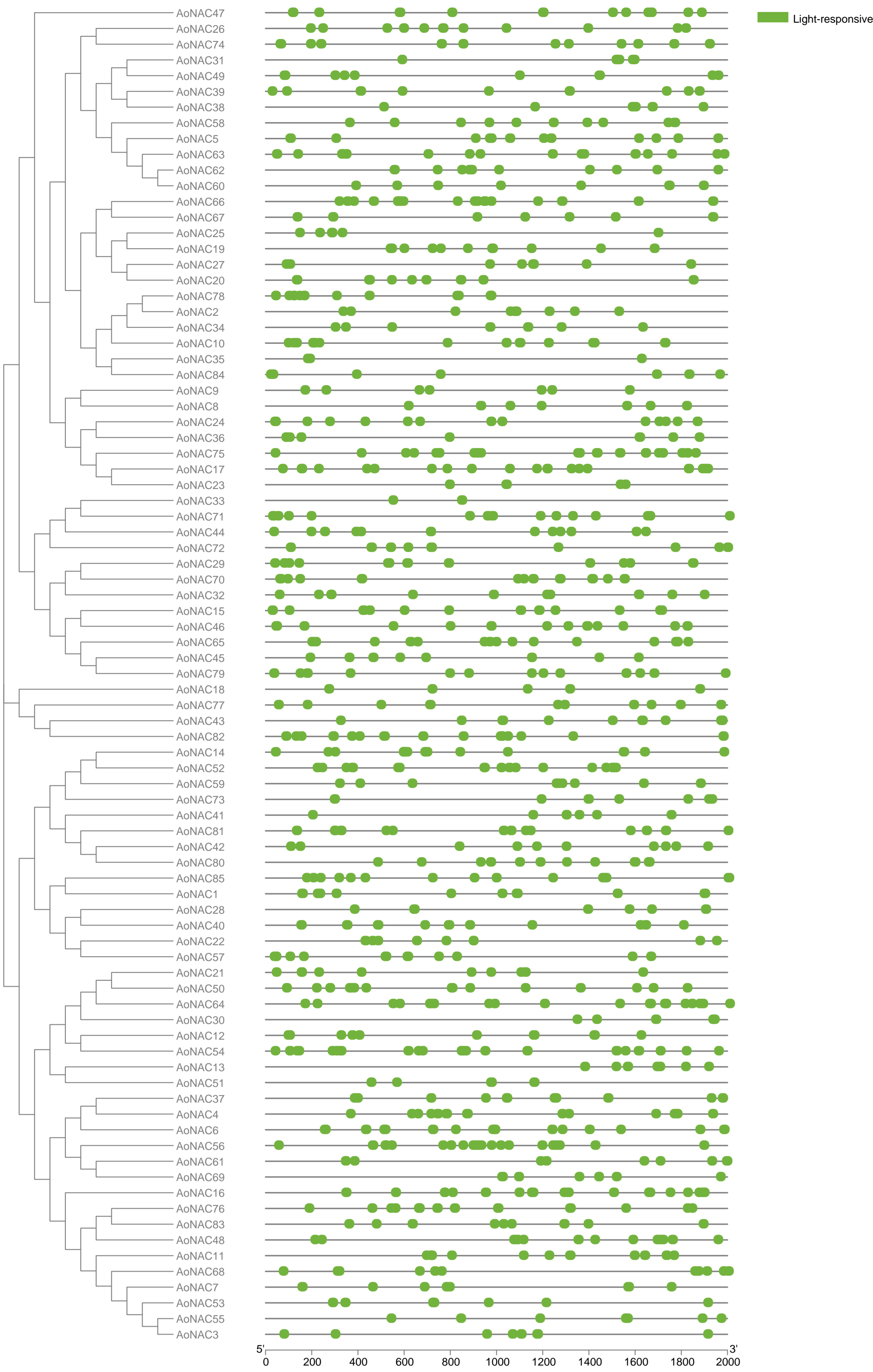

Supplement: Supplementary file 1 [file genes-13-00976-s001.zip › Supplementary Files/Figure S1-Light-responsive element in 85 AoNAC genes.pdf]

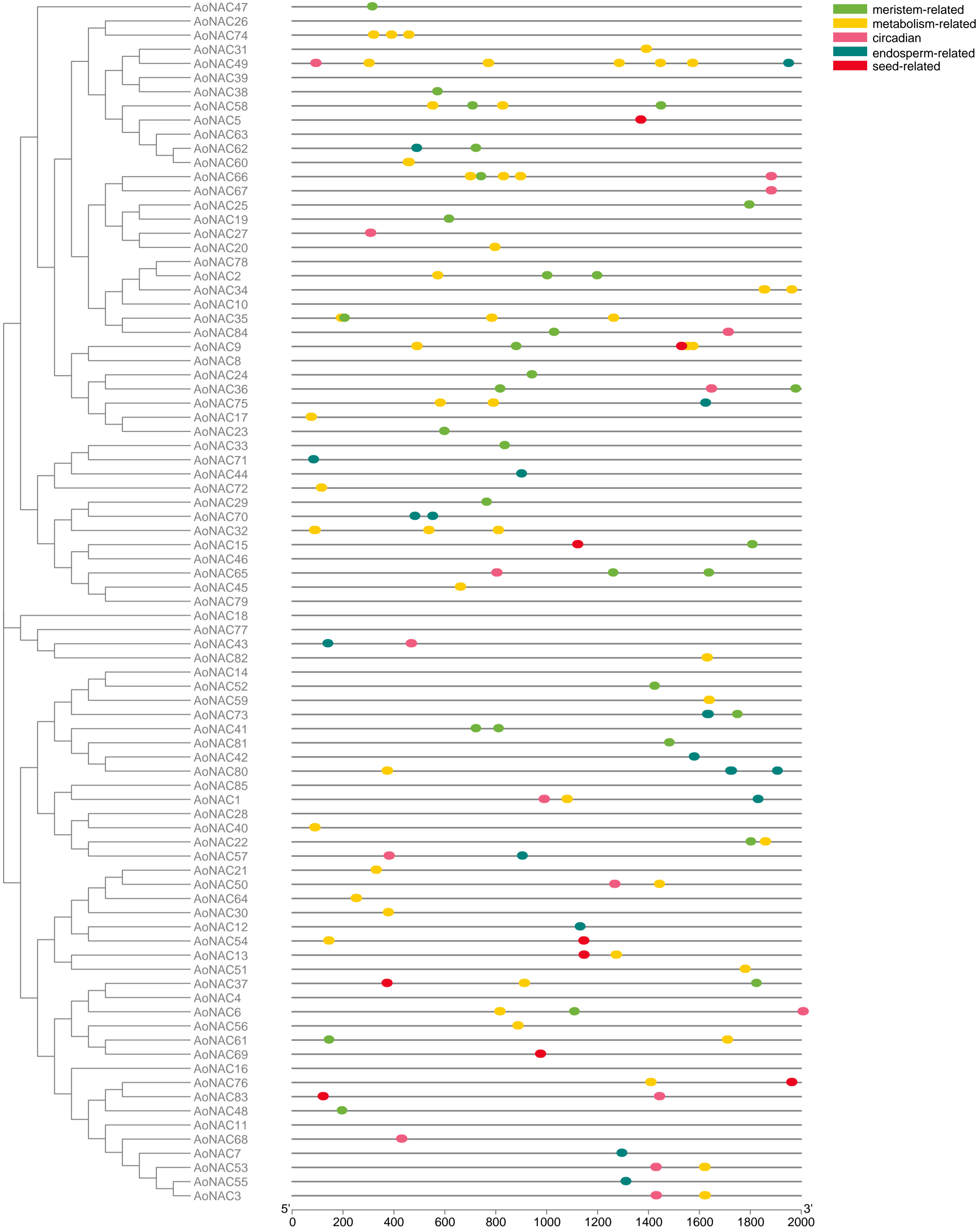

Supplement: Supplementary file 1 [file genes-13-00976-s001.zip › Supplementary Files/Figure S2-Plant growth and development-related element in 69 AoNAC genes.pdf]

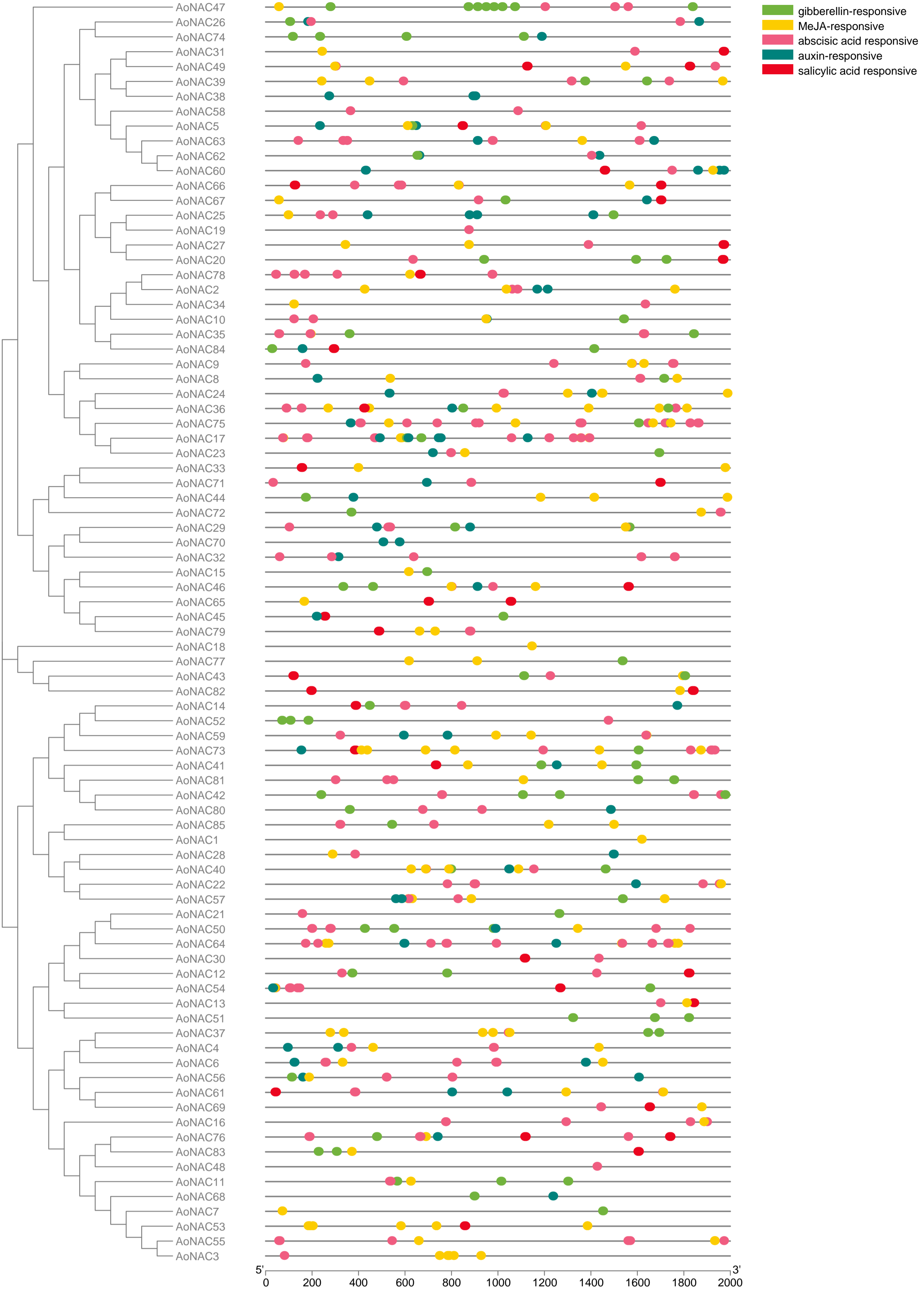

Supplement: Supplementary file 1 [file genes-13-00976-s001.zip › Supplementary Files/Figure S3-Hormone-responsive element in 85 AoNAC genes.pdf]

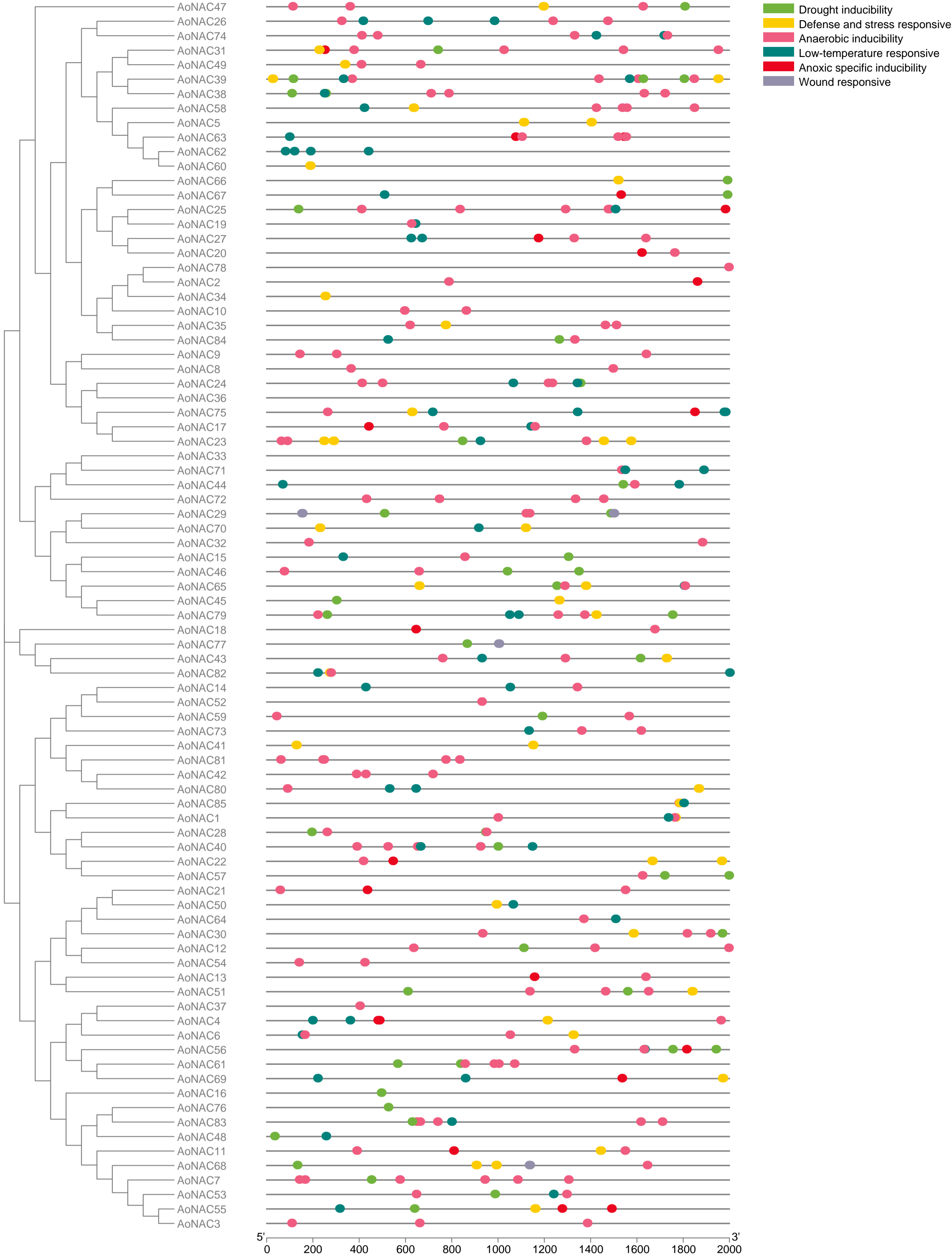

Supplement: Supplementary file 1 [file genes-13-00976-s001.zip › Supplementary Files/Figure S4-Stress-responsive element in 83 AoNAC genes.pdf]
